# Supplementary material for: Content-rich biological network constructed by mining PubMed abstracts
Source: BMC Bioinformatics. 2004 Oct 8;5:147. doi: 10.1186/1471-2105-5-147 (PMC528731; doi:10.1186/1471-2105-5-147)
Supplement: Additional File 2 — The original results of the above study (non-essential files are deleted to keep the file size under the limit set by BMC bioinformatics). [file 1471-2105-5-147-S2.bz2 › chilibotAdditionalFile2/dip05/752ID8393934E18137/html/RUVA_RUVB.html]

 


 **RUVA** and **RUVB** 
  
Found 87 abstracts in PubMed, retrieved 05.  
 

 What does Google say? 
 PDF only 
| .edu only 

---

**Interactive relationship** (e.g. stimulation, inhibition, etc)

**Neutral relationship**- This process involves two key steps branch migration, catalysed by the  **RuvB**  protein that is targeted to the Holliday junction by the structure specific  **RuvA**  protein, and resolution, which is catalysed by the RuvC endonuclease.  Ref: 12423347 Eur J Biochem, 2002
- Two  **RuvA**  tetramers form the symmetric and closed octameric shell, where four  **RuvA**  domain IIIs spring out in the two opposite directions to be individually caught by a single  **RuvB** .  Ref: 12408833 Mol Cell, 2002
- The model of the  **RuvA**   **RuvB**  junction DNA ternary complex, constructed by fitting the X ray structure into the averaged electron microscopic images of the  **RuvA**   **RuvB**  junction, appears to be more compatible with the branch migration mode of a fixed  **RuvA**   **RuvB**  interaction than with a rotational interaction mode.  Ref: 12408833 Mol Cell, 2002
- **RuvA**  has a well defined DNA binding surface that is sculptured specifically to accommodate a Holliday junction and allow subsequent loading of  **RuvB**  and RuvC.  Ref: 11874468 Eur J Biochem, 2002

**Non-interactive relationship** (e.g. studied together, co-existance, homology, etc.)

- Loss of lambda prophage recombinogenicity in UV irradiated Escherichia coli the role of host genes  **ruvA** ,  **ruvB** , ruvC, and recG.  Ref: 11766962 Res Microbiol, 2001
- We present the X ray structure of the  **RuvA**   **RuvB**  complex, which plays a crucial role in ATP dependent branch migration.  Ref: 12408833 Mol Cell, 2002
- Crystal structure of the  **RuvA**   **RuvB**  complex a structural basis for the Holliday junction migrating motor machinery.  Ref: 12408833 Mol Cell, 2002
- Replacements of yacF, yacG, yacH, yacK cueO, yacL,  **ruvA** ,  **ruvB** , yabB, and yabC made with the cassette were used to verify its properties.  Ref: 12142427 J Bacteriol, 2002
- Irradiated prophage retained its ability to recombine in  **ruvA** ,  **ruvB** , ruvC, and recG mutants.  Ref: 11766962 Res Microbiol, 2001
- The  **RuvA** ,  **RuvB**  and RuvC proteins of Escherichia coli act together to process Holliday junctions formed during recombination and DNA repair.  Ref: 11874468 Eur J Biochem, 2002
